# Supplementary material for: Triboelectrification induced self-powered microbial disinfection using nanowire-enhanced localized electric field
Source: Nat Commun. 2021 Jun 17;12:3693. doi: 10.1038/s41467-021-24028-5 (PMC8211783; doi:10.1038/s41467-021-24028-5)
Supplement: Supplementary file 1 — Supplementary information. [file 41467_2021_24028_MOESM1_ESM.pdf]

Supplementary information (SI)

Triboelectrification induced self-powered microbial disinfection using nanowire-enhanced localized electric field

*Zheng-Yang Huo[1, ‡], Young-Jun Kim[1, ‡], In-Yong Suh[1], Dong-Min Lee[1], Jeong Hwan Lee[1], Ye Du[2], Si Wang[1, 3], Hong-Joon Yoon[1], and Sang-Woo Kim[1, 4, 5, *]*

[1]School of Advanced Materials Science and Engineering, Sungkyunkwan University (SKKU), Suwon, 16419, Republic of Korea.

[2]College of Architecture and Environment, Sichuan University, Chengdu, 610065, PR China.

[3]State Key Laboratory of Electronic Thin Films and Integrated Devices, School of Optoelectronic Science and Engineering, University of Electronic Science and Technology of China (UESTC), Chengdu 610054, PR China.

[4]SKKU Advanced Institute of Nanotechnology (SAINT), Sungkyunkwan University (SKKU), Suwon, 16419, Republic of Korea.

[5]Samsung Advanced Institute for Health Sciences & Technology (SAIHST), Sungkyunkwan University (SKKU), Suwon 16419, Republic of Korea.

*Address correspondence to kimsw1@skku.edu

‡ Z.-Y. Huo and Y.-J. Kim contributed equally to this work.

Table of Contents

Figures

Supplementary Fig. 1. Photo of the RV-disinfection system.

Supplementary Fig. 2. SEM of $Cu(OH)_2$ nanowires-modified copper electrode.

Supplementary Fig. 3. SEM and TEM of Cu_3P nanowires-modified copper electrode.

Supplementary Fig. 4. Photo of the V-TENG.

Supplementary Fig. 5. Output voltage of the top and middle layer of the V-TENG.

Supplementary Fig. 6. Output voltage of the TENG with various amplitudes.

Supplementary Fig. 7. Output current of the TENG after rectification.

Supplementary Fig. 8. Structure of the V-TENG-powered RV-disinfection system.

Supplementary Fig. 9. Process of microbial quantification.

Supplementary Fig. 10. Disinfection performance of RV-disinfection with various amplitudes.

Supplementary Fig. 11. Disinfection performance of RV-disinfection for treating the intermittent viral bioaerosols containing MS2.

Supplementary Fig. 12. Disinfection performance of RV-disinfection for treating *E. coli* with different concentrations.

Supplementary Fig. 13. Disinfection performance of RV-disinfection for treating MS2 with different concentrations.

Supplementary Fig. 14. Disinfection performance at various humidity.

Supplementary Fig. 15. Simulation of the contact efficiency of particles after flowing through the negative electrode with different structures (macro-mesh, slope, and column).

Supplementary Fig. 16. XPS and XRD analysis of Cu_3PNWs and NPs-modified electrodes.

Supplementary Fig. 17. Simulation of electric field distribution near the surface of Cu_3PNPs

Supplementary Fig. 18. Biocompatibility tests of the released Cu_3P.

Supplementary Fig. 19. Bulk surface temperatures of the Cu_3PNWs-modified positive electrode during operation.

Tables

Supplementary Table 1. Details for the experiment conditions of air disinfection methods in comparison of Fig. 3h.

Supplementary Table 2. Parameters used for airfield simulation.

Supplementary Table 3. Parameter used for electric field simulation.

Supplementary Notes

Supplementary Note 1. The design of the V-TENG.

Supplementary Note 2. The design of the duct.

Supplementary Note 3. Calculation of microbes trapped by the positive/ground electrode.

Figures:

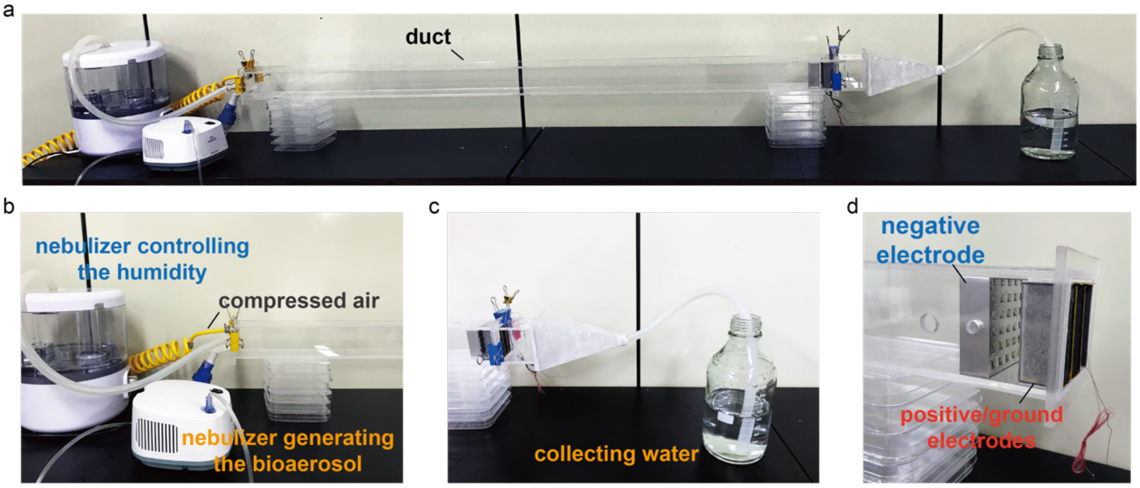

Supplementary Fig. 1 Photo of the RV-disinfection system. **a** Photo showing the whole system of RV-disinfection. **b** Photo showing the bioaerosol generation system as well as the airflow generation and humidity control systems. **c** Photo showing the air collecting system. **d** Photo showing the negative electrode and integrated positive/ground electrodes.

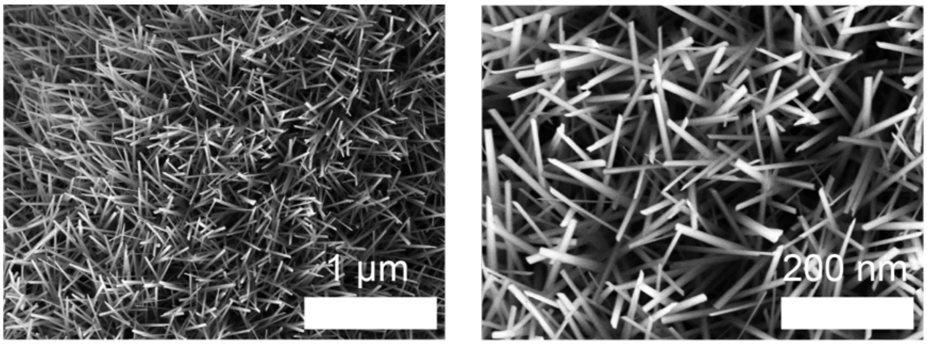

Supplementary Fig. 2 Scanning electron microscope (SEM) images showing the copper hydroxide nanowires-modified copper electrode ($Cu(OH)_2$NW).

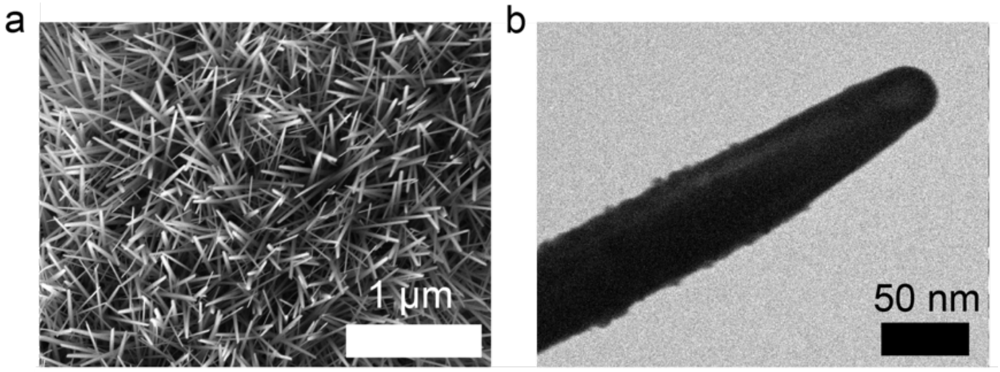

Supplementary Fig. 3 SEM and transmission electron microscope (TEM) images showing the copper-phosphide-nanowire-modified copper electrode (Cu_3PNW-Cu). The TEM measurement was performed using the JEM-2100F from JEOL.

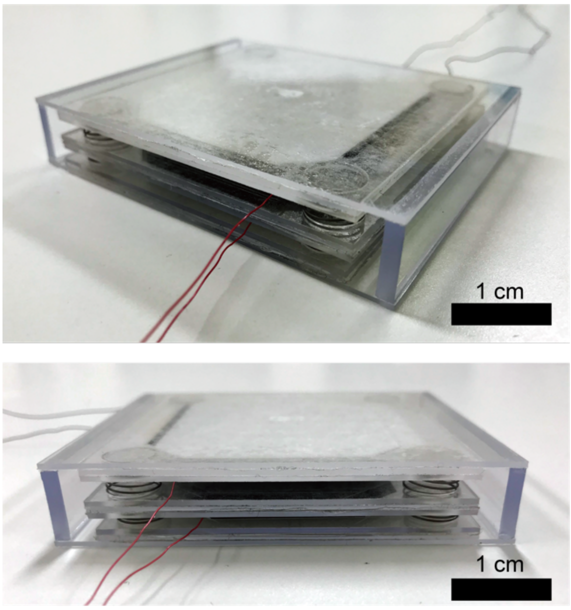

Supplementary Fig. 4 Photo of the V-TENG with three layers (top, middle, and bottom) and a closed structure.

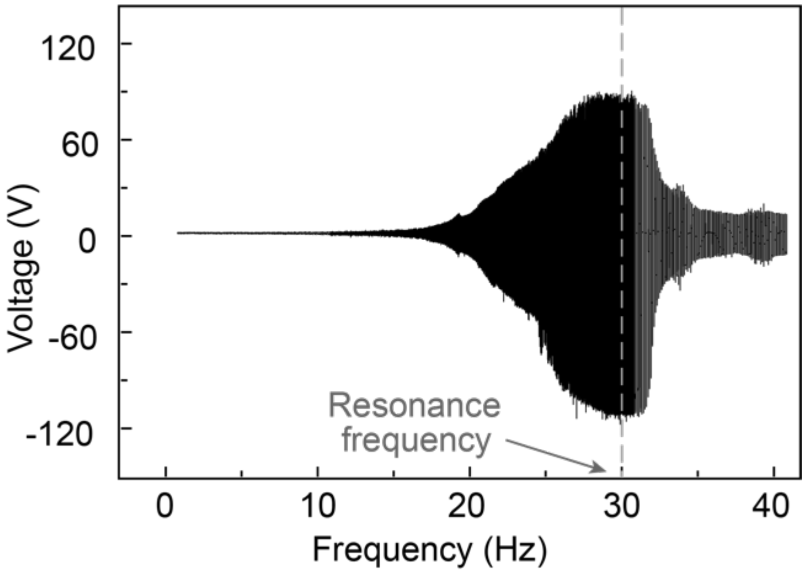

Supplementary Fig. 5 Output voltage of the top and middle layer of the V-TENG with a frequency sweep from 1 Hz to 40 Hz at a constant amplitude of 500 μm. The largest output occurs when the vibration frequency is the same as the resonance frequency of the V-TENG (30 Hz).

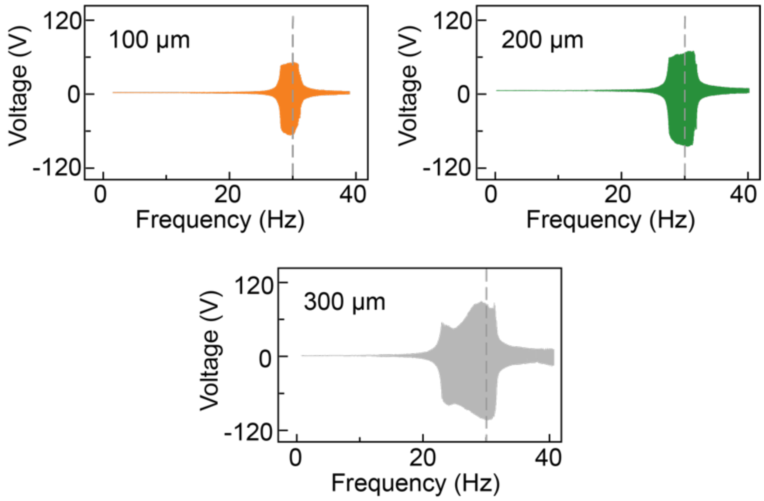

Supplementary Fig. 6 Output voltage of the V-TENG with a frequency sweep from 1 Hz to 40 Hz with various amplitudes (100, 200, and 300 μm). The largest output occurs when the vibration frequency is the same as the resonance frequency of the V-TENG (30 Hz).

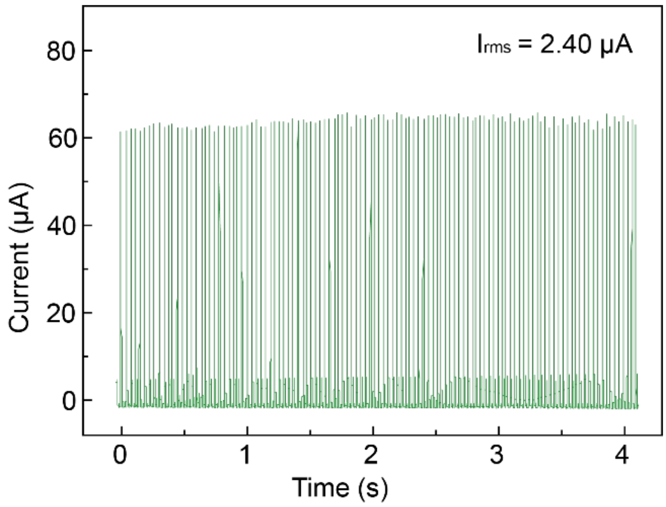

Supplementary Fig. 7 Output current of the V-TENG after rectification at a constant amplitude (500 μm) and frequency (30 Hz).

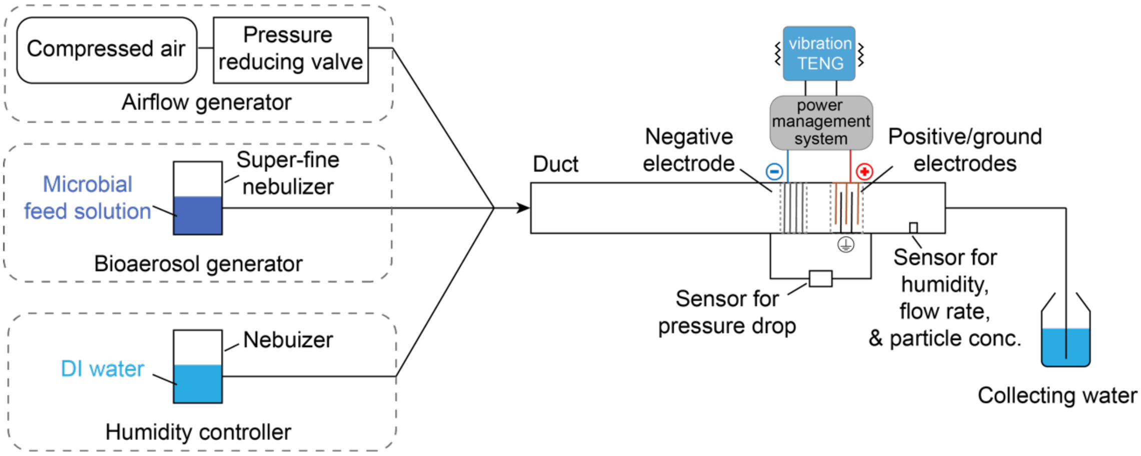

Supplementary Fig. 8 Structure of the V-TENG-powered RV-disinfection system. The feed solutions containing a high concentration of bacteria or viruses were added into a super-fine air compressed nebulizer and bacterial or viral bioaerosols were generated by the nebulizer to feed the duct. Airflow rates in the duct were controlled in the range from 0.5 to 2 m/s using compressed gas and the humidity was set at 30% using another nebulizer generating water mist in the duct. The airflow rate, humidity, and particle concentration were monitored using integrated sensors. After passing through the disinfection filter, the airflow with bacteria or viruses will be collected in a narrow mouth bottle containing 500-mL sterilized deionized (DI) water and the pressure drop will be measured. All the bacteria or viruses will remain in the water for future quantification.

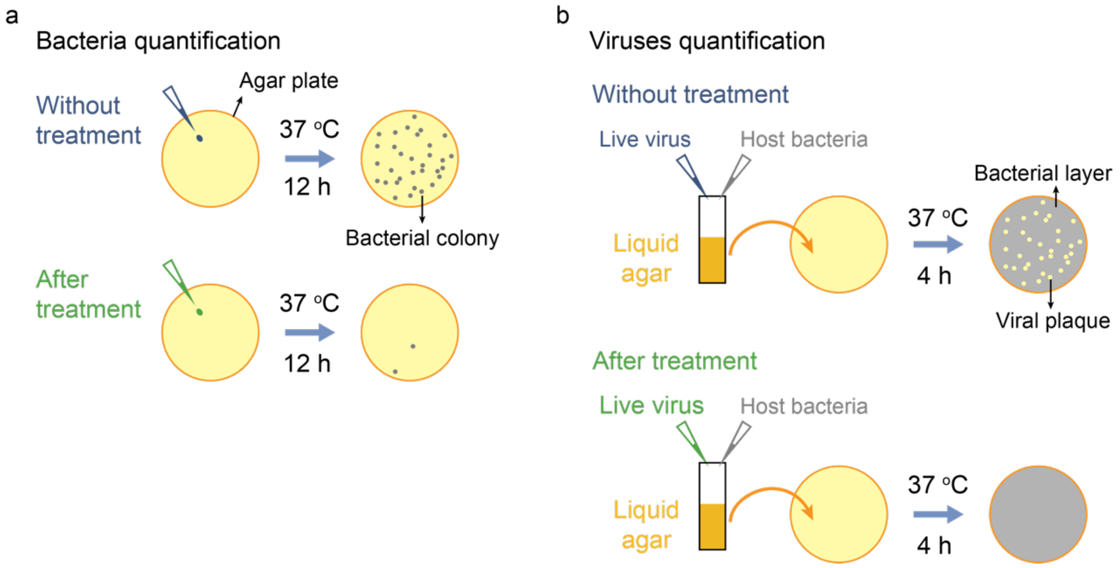

Supplementary Fig. 9 Process of microbial quantification. **a** Schematic of bacteria viability assessment procedures. After the airflow passing through the duct and is collected in the collecting DI water with or without the power from V-TENG, 0.1 mL of collecting water is added to an agar plate and spread evenly using an L-shape spreader. Then put the plate in the incubator at 37 °C for 12 h. Bacterial colonies will present on the agar plate and one bacterial colony represents one single live bacterium. **b** Schematic of virus enumeration procedures. The collecting water (0.1 mL) with viruses is added into a warm liquid agar solution (0.5 mL, ~45 °C). Solution with host bacteria (0.1 mL) will also be added to the liquid agar solution. Then the liquid agar solution will be added to an agar plate. After incubation at 37 °C for 4 h, a thin layer of host bacteria will grow on the agar plate, and if there are live viruses, it will infect and break down the host bacteria and finally form a transparent viral plaque. One viral plaque presenting on the agar plate represents one single live virus. If no bacterial colony or viral plaque was found after incubation, it indicates that all the microbes are inactivated with no live microbes that are detected in the water.

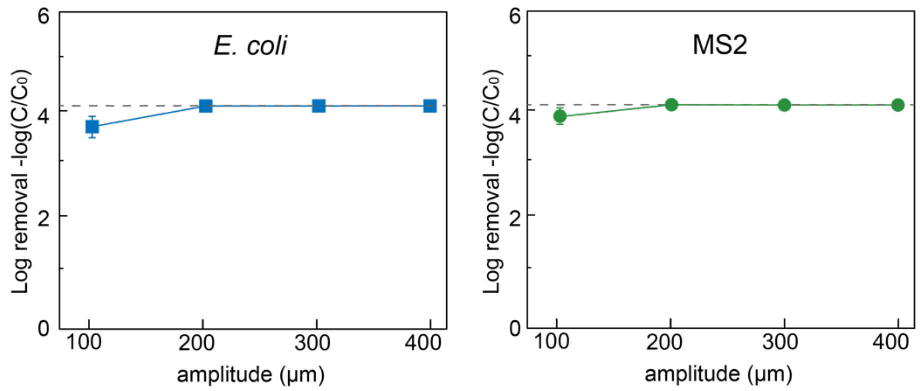

Supplementary Fig. 10 Disinfection efficiency for *E. coli* and MS2 using RV-disinfection methods at various amplitudes (from 100 to 400 μm) and a fixed airflow rate (2 m/s). The dashed line indicates that all microbes (bacteria and viruses) are inactivated and no live microbes can be detected. When applied with smaller amplitudes (100 to 400 μm), the power outputs generated from the V-TENG are sufficient to drive the RV-disinfection system for high-performance disinfection. The error bars represent the standard deviation of three replicate measurements.

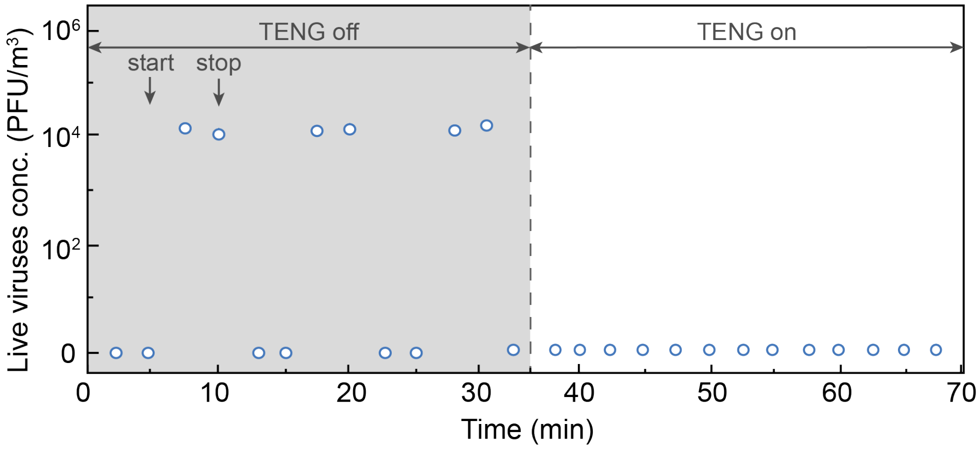

Supplementary Fig. 11 Disinfection performance of the RV-disinfection method for treating the intermittent viral bioaerosols containing MS2. No live viruses can be detected in the intermittent bacterial bioaerosols after passing through the V-TENG powered RV-disinfection system. The V-TENG is operated at a constant amplitude (500 μm) and frequency (30 Hz) and the airflow rate is fixed at 2 m/s.

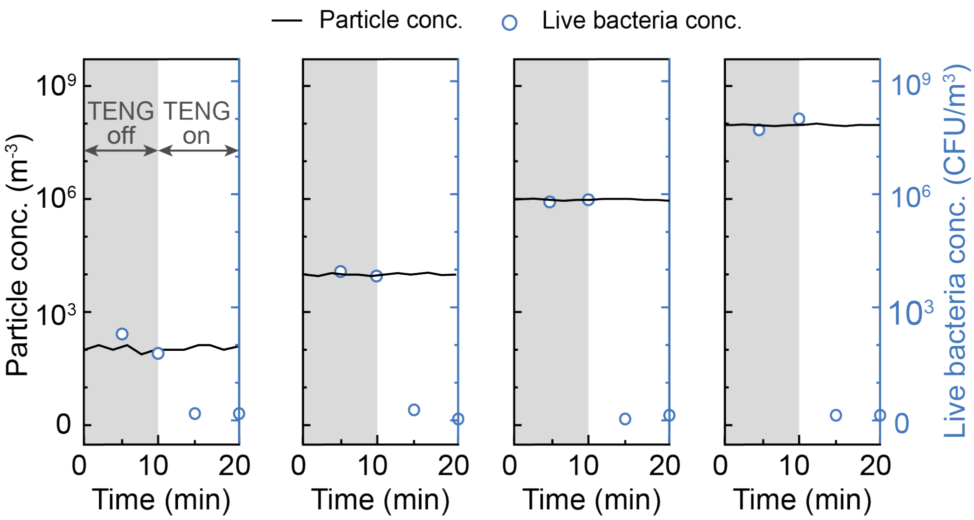

Supplementary Fig. 12 Disinfection performance of the RV-disinfection method for treating *E. coli* with different concentrations (from 10^2 to 10^8 colony-forming unit/m^3, CFU/m^3). No live bacteria can be detected after passing through the V-TENG powered RV-disinfection system. The V-TENG is operated at a constant amplitude (500 μm) and frequency (30 Hz) and the airflow rate is fixed at 2 m/s.

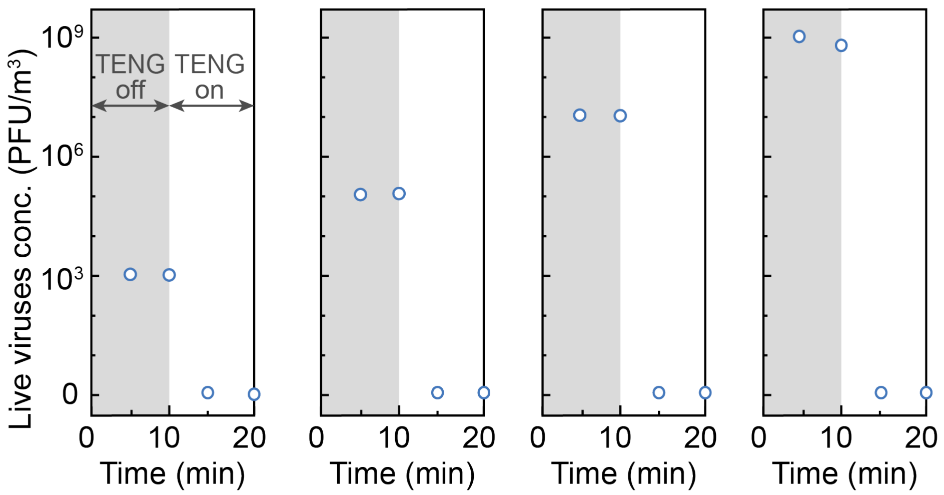

Supplementary Fig. 13 Disinfection performance of the RV-disinfection method for treating MS2 with different concentrations (from 10^3 to 10^9 plaque-forming unit/m^3, PFU/m^3). No live virus can be detected after passing through the V-TENG powered RV-disinfection system. The V-TENG is operated at a constant amplitude (500 μm) and frequency (30 Hz) and the airflow rate is fixed at 2 m/s.

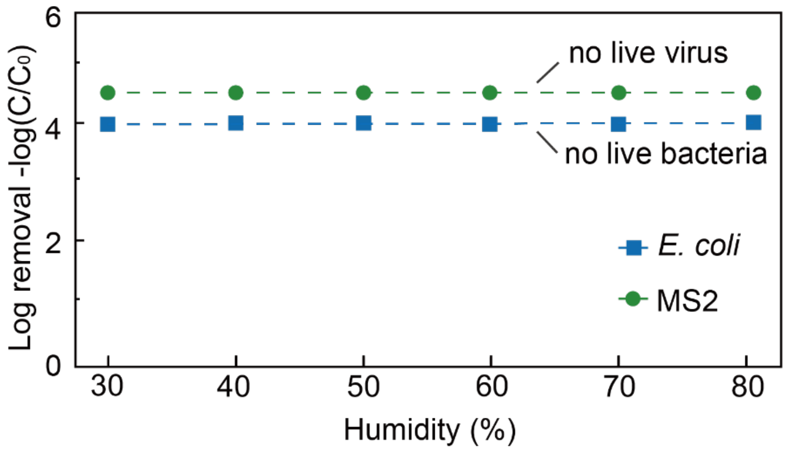

Supplementary Fig. 14 Disinfection performance at various humidity. Feed solutions containing a high concentration of bacteria (*E. coli*) or viruses (MS2) were added into a super-fine air compressed nebulizer and bacterial or viral bioaerosols were generated by the nebulizer to flow through the duct. The initial concentration of MS2 is relatively higher than that of *E. coli*. Thus, when the microbes are completely inactivated, the log removal efficiency of MS2 (> 4.4-log) and *E. coli* (> 4.0-log) will be different. The airflow rate in the duct was fixed at 2 m/s using compressed gas. The humidity was controlled from 30% to 80% using another nebulizer to generate water aerosols in the duct. Dashed lines indicate that all microbes are inactivated, and no live microbes can be detected. All the tested bacteria (*E. coli*) and viruses (MS2) can be inactivated completely in a wide range of humidity (from 30% to 80%) at a fast airflow rate.

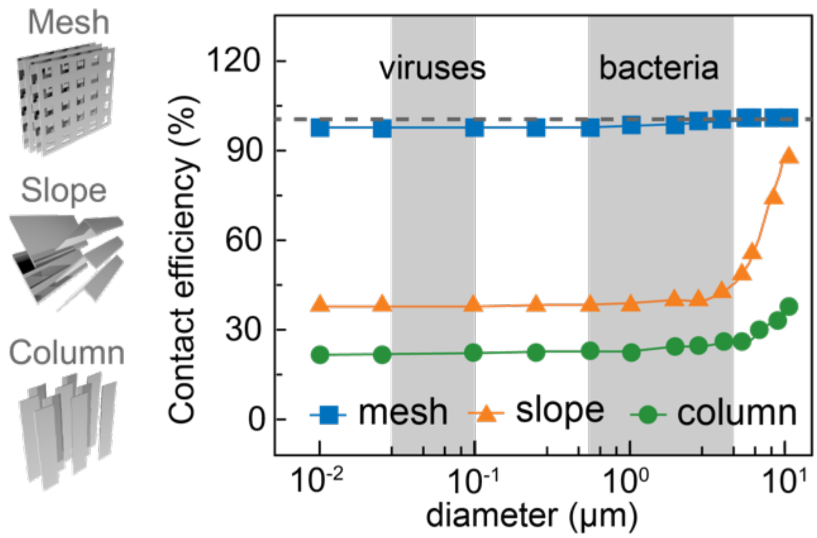

Supplementary Fig. 15 Simulation of the electrode's contact efficiency (percentage of particles contacting the electrode) after flowing through the 4-layer electrode with different structures (macro-mesh, slope, and column). The dashed line indicates 100% contact efficiency. The macro-mesh structure is confirmed to be the ideal electrode structure due to it giving the highest contact efficiency compared to other structures (slope and column).

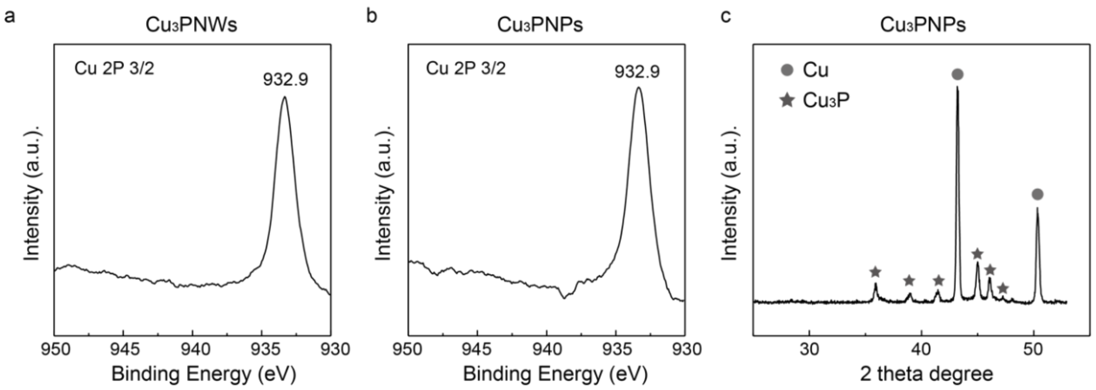

Supplementary Fig. 16 X-ray photoelectron spectroscopy (XPS) and X-ray diffraction (XRD) analysis of Cu_3P nanowires and nanoparticles-modified electrodes confirming formations of Cu_3P for both NWs and NPs samples. (a and b) Cu *2p* spectra of the Cu_3PNWs (a) and Cu_3PNPs (b) -modified copper electrodes. The major peaks shown in both Cu_3PNWs and Cu_3PNPs samples at 932.9 eV for the Cu $2p_{3/2}$ energy level are attributed to $Cu^{\delta+}$ in Cu_3P [1]. (c) XRD pattern of the Cu_3PNPs-modified copper electrode. Besides the two strong diffraction peaks at 43.4° and 50.6° from the Cu substrate (JCPDS file No. 04-0836), the other peaks can be assigned to the Cu_3P phase. The diffraction pattern for the Cu_3PNPs-modified copper electrode exhibits six peaks at 36.0°, 39.1°, 41.6°, 45.1°, 46.2°, and 47.3°, corresponding to (112), (202), (211), (300), (113), and (212) of Cu_3P phase (JCPDS file No. 71-2261), respectively [2]. a.u., arbitrary units.

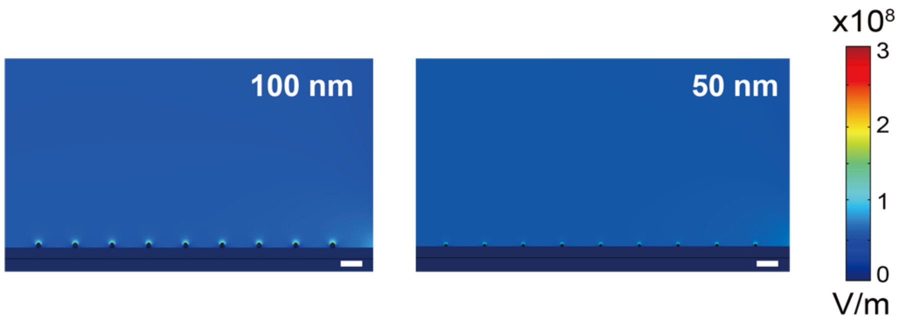

Supplementary Fig. 17 Simulation of electric field distribution near the surface of Cu_3PNPs (diameter of 100 and 50 nm) driven by the V-TENG, showing the relatively weak enhancement of the localized electric field. The scale bar is 500 nm.

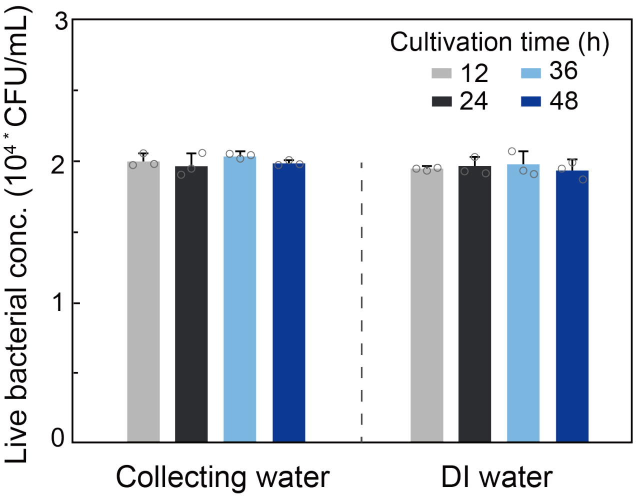

Supplementary Fig. 18 Biocompatibility tests of the released Cu_3P. Air (1 m^3) after flowing through the disinfection device was collected in sterilized DI water (500 mL). Bacterial solution (1 mL; 10^7 CFU/mL) was fed in the collecting water and cultivated at a fixed temperature (25 °C) for 48 h. During the cultivation process, the concentration of the live bacteria was investigated. To make a comparison, another 1 mL of bacteria solution (10^7 CFU/mL) was added in DI water to investigate the concentration of the live bacteria with the same operating condition. During the cultivation process, the concentration of live bacteria in both collecting water with Cu_3P and DI water (control sample) was similar and didn't decrease. This confirmed that the released Cu_3P was ineffective for bacterial inactivation. The toxicity of the collecting water is low due to the low concentration of the released Cu_3P. The error bars represent the standard deviation of three replicate measurements.

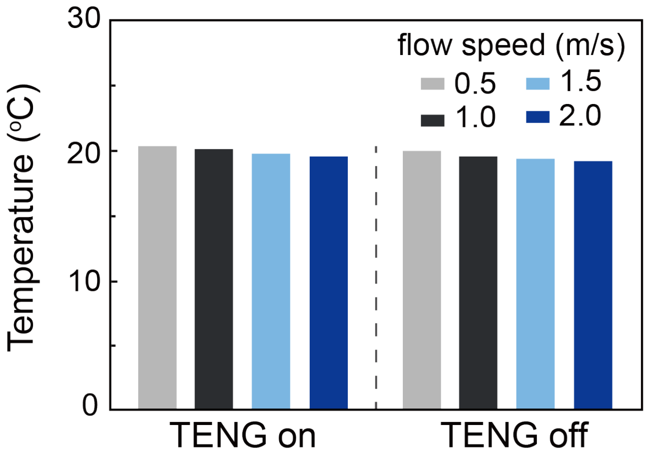

Supplementary Fig. 19 Bulk surface temperatures of the Cu_3PNWs-modified positive electrode with or without the power from TENG for 30 min. The humidity was fixed at 30% with airflow rates ranging from 0.5 to 2.0 m/s. No microbes were applied in this experiment. No significant temperature fluctuation of the electrode occurred during the air disinfection, confirming little contribution of Joule heating to microbial inactivation.

Tables:

Supplementary Table 1. Details for the experiment conditions of air disinfection methods in comparison of Fig. 3h (materials, disinfection mechanisms, energy demands, and bacteria strain).

Materials	Mechanisms	Energy demands	Microbial strain	Ref.
Cu_3PNW-Cu	Electroporation	Self-powered	*E. coli, B. subtilis,* & MS2	This work
MOF-filter	Photocatalytic	Sunlight	*E. coli*	3
TiO_2-film	Photocatalytic	Ultraviolet radiation	*E. coli*	4
ZnO-Al_2O_3-filter	Antibacterial nanomaterial	No	*B. atrophaeus*	5
Fe_2O_3NW-Fe mesh	Electroporation	Electricity	*E. coli & S. epidermidis*	6
Vacuum UV	UV disinfection	Electricity	*E. coli*	7
UV-LED	UV disinfection	Electricity	*E. coli*, *S. Typhimurium*, *S. aureus*, & MS2	8

Supplementary Table 2. Parameters used for airfield simulation.

Parameter	Unit	Value
Airflow rate	m/s	2
Length of the duct	m	1.4
Cross area of the duct	cm × cm	6 × 6
Cross area of the negative electrode	cm × cm	6 × 6
Thickness of the negative electrode	mm	1
Size of the pore on the negative electrode	cm × cm	5 × 5
Distance between different layers	mm	3

Supplementary Table 3. Parameters used for electric field simulation.

Parameter	Unit	Value
Size of the electrode	cm × cm	6 × 2
Distance between two electrodes	cm	1
Density of the nanowire	μm^{-2}	9
Distance between two nanowires	μm	0.5
Length of the nanowire	μm	5
Diameter of the nanowire	nm	50
Electric potential	V	100
Vacuum permittivity	F/m	8.85E-12 [9]
Relative Permittivity of air (ε_f)	1	1.0006 [9]

Supplementary Notes:

Supplementary Note 1. The design of the V-TENG.

To ensure the high output of the V-TENG, the resonance frequency of the middle layer of the V-TENG was designed the same as the common operation frequency of the ventilator of the indoor building.

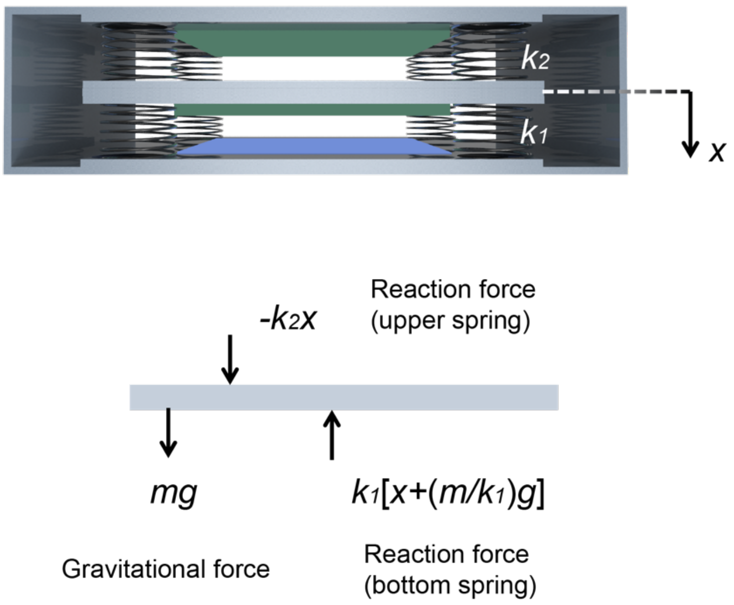

Supplementary Fig. 20 Schematic showing the structure of the V-TENG.

As the structure shown in Supplementary Fig. 20, the middle layer with the mass of *m* (g) was supported by the springs with a constant of k (N/m). The displacement of the middle layer during the vibration is *x* (m) and the acceleration of the middle layer is *a*. The movement can be described according to the following equation (Eq. 1).

$$mg - \mathrm{k}_2 x - \mathrm{k}_1\left(x + \frac{m}{\mathrm{k}_1}\mathrm{g}\right) = ma \qquad (1)$$

Since acceleration and displacement are functions of time and if we assume that the remaining variables are constant, we can get a homogeneous linear ordinary differential equation with constant coefficients (Eq. 2):

$$\ddot{x} + \frac{(\mathrm{k}_1 + \mathrm{k}_1)}{m}x = 0 \qquad (2)$$

The general solution of this differential equation can be expressed with the following equation (Eq. 3):

$$x = \mathrm{A}\sin(\omega t) + \mathrm{B}\cos(\omega t) \qquad (3)$$

where ω is the resonance frequency of the system, A is the variable, and B can be determined by the boundary conditions of the system. Thus the ω can be determined as shown in Eq. 4 [10].

$$\omega = \frac{1}{2\pi}\sqrt{\frac{\mathrm{k}_1+\mathrm{k}_2}{m}} \qquad (4)$$

When $k_1 = k_2 = 4 * 82.712 = 330.84$ N/m (each spring with a constant of 82.712 N/m) and m = 0.02 kg the ω can be calculated as 28.95 Hz. The highest output can be obtained near the resonance frequency since the displacement of the middle layer can be maximized.

Supplementary Note 2. The design of the duct.

The duct was designed according to the practical application of the ventilation system of indoor buildings. The parameters of the duct and the operating conditions were shown in Supplementary Table 2 and the structure of the duct as shown in Supplementary Fig. 21.

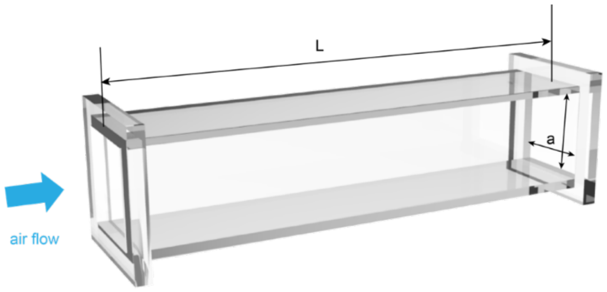

Supplementary Fig. 21 Schematic showing the duct.

To make sure the airflow in the duct is laminar flow and decrease the impact of mass transfer introduced by the turbulence, the length of the duct was design based on the following equations (Eq. 5 to 8):

$$Re = \rho v D/\mu \qquad (5)$$

$$D = 4A/p \qquad (6)$$

$$A = a^2 \qquad (7)$$

$$p = 4a \qquad (8)$$

where Re is the Reynolds number, ρ is the density of the air (1.2 kg/m^3), v is the airflow rate (2 m/s), D is the characteristic length of the duct, A is the area of the cross area of the duct, a is the width of the duct (6 cm), p is the length of the surrounding flow, and μ is the viscosity of the air (1.8 × 10^{-5} kg/(m·s). The Re number was calculated as 7955.8 which is in the range of turbulence flow. To ensure the laminar flow in the duct, the length (L) was calculated based on the following equation (Eq. 9):

$$L = 4.4D \times Re^{\frac{1}{6}} \qquad (9)$$

and the length of the duct was calculated as 1.18 m. Thus, in this study, the length of the duct was designed as 1.4 m to ensure the inside laminar flow.

Supplementary Note 3. Calculation of microbes trapped by the positive/ground electrode.

When the negatively charged microbes flow through the positive/ground integrated electrodes, they will be trapped on the positive electrode surface by the electrostatic attraction. The schematic in Supplementary Fig. 22 showed the microbes flowing in between the positive/ground integrated electrodes.

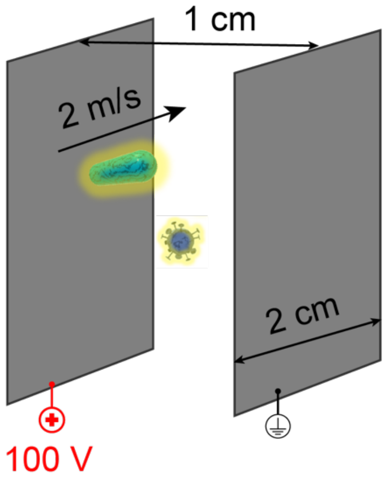

Supplementary Fig. 22 Schematic showing the microbes flowing in between the positive/ground integrated electrodes.

The time for microbes approaching the positive electrode surface (t) will be quantified based on the following equations (Eq. 10 to 12):

$$D = \frac{1}{2}at^2 \qquad (10)$$

$$a = \frac{F}{m} \qquad (11)$$

$$F = E \times q \qquad (12)$$

where D is the distance between the positive and ground electrode (1 cm), a is the acceleration of the microbes in the horizontal direction, F is the force on the microbes in the horizontal direction, which is the force of electrostatic attraction, m is the mass of the microbes (10^{-12} and 10^{-18} g for bacterium and virus) [11, 12], E is the electric field strength between the positive and ground electrode (10^4 V/m), and q is the charges carried by the microbes (6.1×10^{-10} and $7.2\times10^{-}$

^{12} C for bacterium and virus). When considering these parameters, the time for microbes approaching the positive electrode surface was calculated ~10^{-5} and ~10^{-7} s, respectively, which is significantly smaller than the time needed for airflow passing through the electrode (2 cm ÷ 2 m/s = 0.01 s). Thus, attributed to the charging of microbes, the microbes can be trapped to the positive electrode surface effectively, ensuring disinfection efficiency.

If considering the air resistance during the trapping process, due to the microbes move in the laminar flow, the air resistance (F_d) can be described as the following equation (Eq. 13):

$$F_d = 6\pi\mu r V \qquad (13)$$

where r is the radius of the microbes, μ is the viscosity of the air (1.8 × 10^{-5} kg/(m·s), and V is the speed of the microbes. The possible highest speed can be calculated as follows (Eq. 14):

$$V = a \times t \qquad (14)$$

where a is the acceleration caused by the force of electrostatic attraction (6×10^{6} m/s^2), and t is the movement time (~10^{-5} s). So, the V can be calculated as 60 m/s and the F_d for the bacteria was 10^{-11} N, which is significantly lower than the force of electrostatic attraction (F=m×a; 10^{-6} N). Thus, the air resistance will not impact the trapping time.

Supplementary Reference:

[1] Fan, Mouping, et al. "Half-cell and full-cell applications of highly stable and binder-free sodium ion batteries based on Cu_3P nanowire anodes." Advanced Functional Materials 26.28 (2016): 5019-5027.
[2] Huo, Zheng-Yang, et al. "A Cu_3P nanowire enabling high-efficiency, reliable, and energy-efficient low-voltage electroporation-inactivation of pathogens in water." Journal of Materials Chemistry A 6.39 (2018): 18813-18820.
[3] Li, Ping, et al. "Metal-organic frameworks with photocatalytic bactericidal activity for integrated air cleaning." Nature Communications 10.1 (2019): 1-10.
[4] Sánchez, Benigno, et al. "Photocatalytic elimination of indoor air biological and chemical pollution in realistic conditions." Chemosphere 87.6 (2012): 625-630.
[5] Dutheil de la Rochère, Aliénor, et al. "Exploring the antimicrobial properties of dark-operating ceramic-based nanocomposite materials for the disinfection of indoor air." PloS one 14.10 (2019): e0224114.
[6] Wang, Dawei, et al. "Iron oxide nanowire-based filter for inactivation of airborne bacteria." Environmental Science: Nano 5.5 (2018): 1096-1106.
[7] Huang, Haibao, et al. "Photocatalytic destruction of air pollutants with vacuum ultraviolet (VUV) irradiation." Catalysis Today 175.1 (2011): 310-315.
[8] Kim, Do-Kyun, and Dong-Hyun Kang. "UVC LED irradiation effectively inactivates aerosolized viruses, bacteria, and fungi in a chamber-type air disinfection system." Applied and Environmental Microbiology 84.17 (2018).
[9] Lide, David R., ed. CRC handbook of chemistry and physics. Vol. 85. CRC press, 2004.
[10] Chen, Jun, et al. "Harmonic-resonator-based triboelectric nanogenerator as a sustainable power source and a self-powered active vibration sensor." Advanced materials 25.42 (2013): 6094-6099.
[11] The database of ecmdb: E. Coli Numbers and Stats - https://ecmdb.ca/e_coli_stats
[12] Kuzmanovic, Deborah A., et al. "Bacteriophage MS2: molecular weight and spatial distribution of the protein and RNA components by small-angle neutron scattering and virus counting." Structure 11.11 (2003): 1339-1348.
